# Identifying factors that indicate the possibility of non-visible cases on mammograms using mammary gland content ratio estimated by artificial intelligence

**DOI:** 10.3389/fonc.2024.1255109

**Published:** 2024-03-05

**Authors:** Chiharu Kai, Tsunehiro Otsuka, Miyako Nara, Satoshi Kondo, Hitoshi Futamura, Naoki Kodama, Satoshi Kasai

**Affiliations:** ^1^ Department of Radiological Technology, Faculty of Medical Technology, Niigata University of Health and Welfare, Niigata, Niigata, Japan; ^2^ Major in Health and Welfare, Graduate School of Niigata University of Health and Welfare, Niigata, Niigata, Japan; ^3^ Otsuka Breastcare Clinic, Tokyo, Japan; ^4^ Department of Breast Surgery, Tokyo Metropolitan Cancer and Infectious Disease Center, Komagome Hospital, Tokyo, Japan; ^5^ Graduate School of Engineering, Muroran Institute of Technology, Muroran, Hokkaido, Japan; ^6^ Healthcare Business Headquarters, Konica Minolta, Inc., Tokyo, Japan

**Keywords:** mammogram, mammary gland content ratio, breast cancer, artificial intelligence, non-visible

## Abstract

**Background:**

Mammography is the modality of choice for breast cancer screening. However, some cases of breast cancer have been diagnosed through ultrasonography alone with no or benign findings on mammography (hereby referred to as non-visibles). Therefore, this study aimed to identify factors that indicate the possibility of non-visibles based on the mammary gland content ratio estimated using artificial intelligence (AI) by patient age and compressed breast thickness (CBT).

**Methods:**

We used AI previously developed by us to estimate the mammary gland content ratio and quantitatively analyze 26,232 controls and 150 non-visibles. First, we evaluated divergence trends between controls and non-visibles based on the average estimated mammary gland content ratio to ensure the importance of analysis by age and CBT. Next, we evaluated the possibility that mammary gland content ratio ≥50% groups affect the divergence between controls and non-visibles to specifically identify factors that indicate the possibility of non-visibles. The images were classified into two groups for the estimated mammary gland content ratios with a threshold of 50%, and logistic regression analysis was performed between controls and non-visibles.

**Results:**

The average estimated mammary gland content ratio was significantly higher in non-visibles than in controls when the overall sample, the patient age was ≥40 years and the CBT was ≥40 mm (p < 0.05). The differences in the average estimated mammary gland content ratios in the controls and non-visibles for the overall sample was 7.54%, the differences in patients aged 40–49, 50–59, and ≥60 years were 6.20%, 7.48%, and 4.78%, respectively, and the differences in those with a CBT of 40–49, 50–59, and ≥60 mm were 6.67%, 9.71%, and 16.13%, respectively. In evaluating mammary gland content ratio ≥50% groups, we also found positive correlations for non-visibles when controls were used as the baseline for the overall sample, in patients aged 40–59 years, and in those with a CBT ≥40 mm (p < 0.05). The corresponding odds ratios were ≥2.20, with a maximum value of 4.36.

**Conclusion:**

The study findings highlight an estimated mammary gland content ratio of ≥50% in patients aged 40–59 years or in those with ≥40 mm CBT could be indicative factors for non-visibles.

## 1 Introduction

Breast cancer is common among women worldwide (1–3). The number of brhavet cancer cases and deaths in Japan has been increasing (3). Early detection of breast cancer can contribute to a higher 10-year survival rate. Therefore, regular screening is critical for the early detection of breast cancer and the initiation of treatment before the appearance of subjective symptoms. Mammography is the national recommendation for breast cancer screening and is the only testing modality that can help reduce mortality (4–7). However, both normal and pathological breast tissues appear as bright lesions on mammography, and cancer lesions may be missed in cases of a high volume of mammary tissue (described as a "dense breast") (8, 9). Asian women, including Japanese women, have denser breasts than those of Western women. A mammary gland content ratio has been evaluated to determine the risk of hidden breast cancers. According to the Japanese guidelines for determining breast composition, it is the area of the mammary gland equal to or greater than the density of the pectoralis muscle divided by the area in the breast where mammary tissues are thought to be present (10). These guidelines are based on the “Breast Imaging Reporting and Data System” (BI-RADS) atlas (11).

Another method to effectively detect cancers in dense breasts is ultrasonography combined with mammography (12, 13). Ultrasonography renders normal breast tissues bright and abnormal lesions dark, helping clinicians to easily distinguish between normal breast tissue and lesions. In fact, breast cancer has also been detected based on ultrasonographic findings alone when mammography showed no or benign findings; cases with non-visible findings on mammograms are hereafter referred to as non-visibles. Identifying such cases on mammograms could contribute to the earlier detection of breast cancer by sending those patients to other examinations, such as ultrasonography.

With the above background, we hypothesized that the mammary gland content ratio differs between healthy individuals (hereafter referred to as controls) and those with non-visibles. As the volume of mammary tissue varies with age (14), and the sensitivity for detecting abnormal lesions is related to compressed breast thickness (CBT) (15, 16), we also hypothesized that age and CBT are related to the mammary gland content ratio in controls and non-visibles. Owing to the need for a tool to evaluate large data volumes, we developed an artificial intelligence (AI) system to estimate the mammary gland content ratio as a continuous value on mammograms (17). We had previously found a high correlation between the mammary gland content ratio generated by AI and that by a specialist (17). The strength of the AI-generated mammary gland content ratio is that it is reproducible and quantifiable, making it suitable for the evaluation of extensive data. Therefore, this study aimed to use a large dataset to identify the factors that indicate the possibility of non-visibles using AI based on age and CBT.

## 2 Materials and methods

The Institutional Review Board of the Niigata University of Health and Welfare approved this study (Approval No. 19010-230303).

### 2.1 Data selection

**Figure 1:**
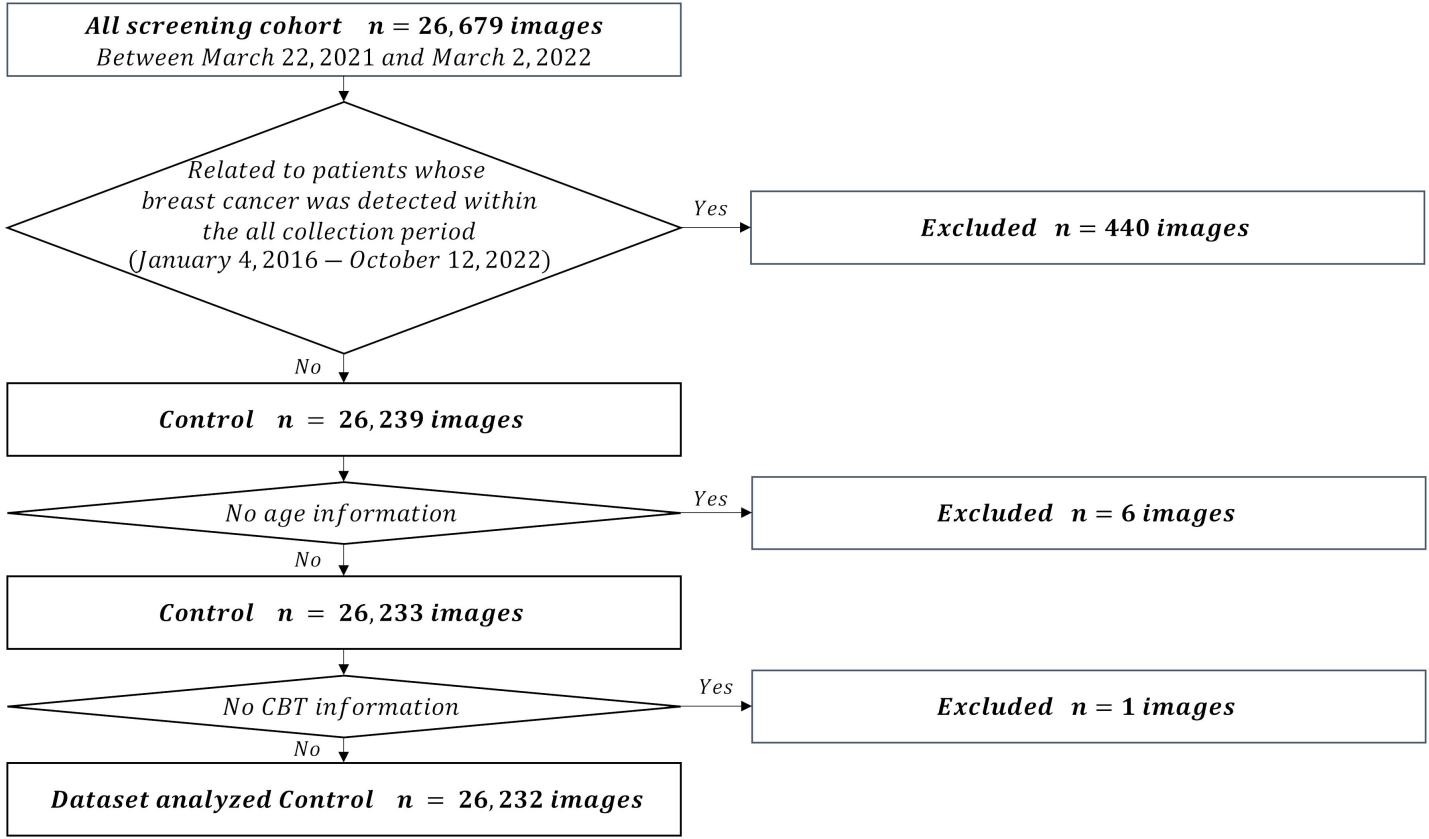
Flowchart for the control dataset.

**Figure 2:**
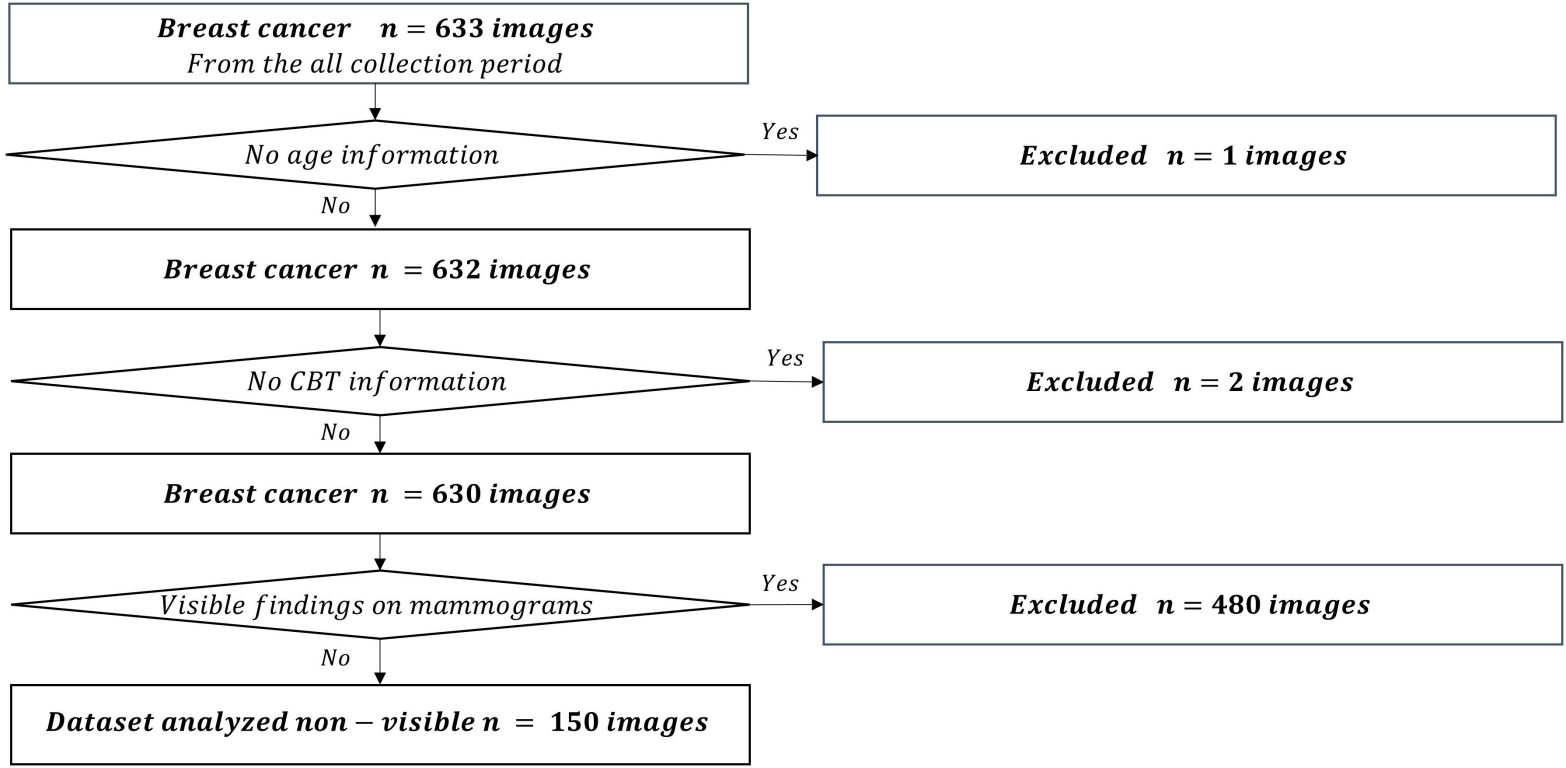
Flowchart for the non-visible dataset.

We used the mediolateral oblique view and determined the mammogram findings according to the Japanese mammography guidelines (10) based on the BI-RADS atlas (11). The dataset used in this study comprised 211,897 mammograms obtained at Otsuka Breastcare Clinic between January 4, 2016, and October 12, 2022. In these images, we collected 26,679 mammograms obtained between March 22, 2021, and March 2, 2022, in the control group, and 633 mammograms obtained during the whole collection period (between January 4, 2016 and October 12, 2022) in the breast cancer group. The choice of the timeframe for the control group was random. These images of breast cancer were confirmed based on histopathological confirmation of cancer diagnosis. Of the 26,679 mammograms in the control group, we excluded a total of 440 mammograms in patients with breast cancer detected during the whole collection period. We excluded six images with no age information and an image with no CBT information. We finally included 26,232 controls (**Figure 1**). Of the 633 breast cancer images, we excluded an image with no age information and two images with no CBT information, consequently totaling 630 breast cancer images. We also excluded 480 images of lesions diagnosed as malignant based on the medical records; images with visible findings on mammograms (as opposed to non-visibles), and finally included 150 non-visibles (**Figure 2**). Those 150 non-visibles were detected on ultrasound and/or visual palpation examination during breast cancer screening or routine practice. We finally included 26,232 controls and 150 non-visibles. Approximately 23.8% (150/630) of all breast cancer cases were non-visibles, which is similar to the 77.0% sensitivity of mammography reported by Ohuchi et al. (12). The mammograms (Pe·ru·ru DIGITAL, Canon Medical Systems Corporation, Tochigi, Japan) used in this study were collected by Konica Minolta and were shared as anonymously processed information. However, Konica Minolta played no role in the study design, analysis, model development, or manuscript preparation.

### 2.2 AI-estimated factors and subgroup determination

**Figure 3:**
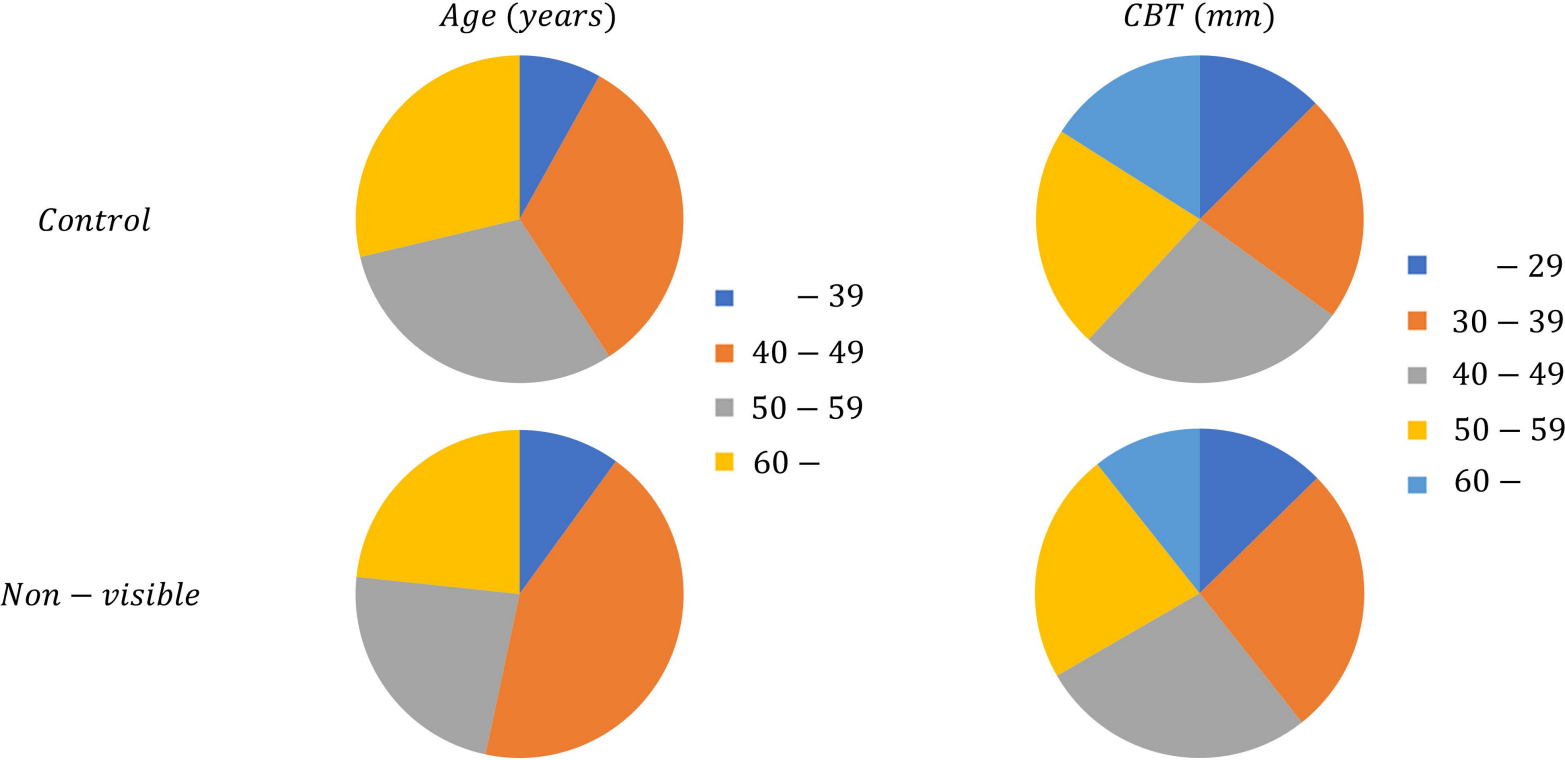
The dataset composition of controls and non-visibles by age and CBT.

We applied the mammary gland content ratio estimated using AI previously developed by us to the controls and non-visibles. We entered the mammograms into this AI, after which the calculated mammary gland content ratios were used in this experiment. We then assessed the divergence in the estimated mammary gland content ratio between the controls and the non-visibles by age (≤39, 40–49, 50–59, and ≥60 years) and CBT (≤29, 30–39, 40–49, 50–59, and ≥60 mm) subgroups. **Table 1** presents the breakdown of the dataset. **Figure 3** shows the characteristics of the control and non-visible groups based on age and CBT. No significant trend was observed in the composition of the dataset based on the CBT. However, the age-specific dataset showed a higher proportion of non-visibles in the 40–49-year group as compared to controls.

**Table 1:** Characteristics of the control and non-visible groups.

	Control	Non-Visible
Images	26,232	150
Date	March 2021 - March 2022	August 2016 - October 2022
Age (years)	18 - 90	30 - 87
	[54.00 ± 12.10]	[51.74 ± 12.83]
CBT (mm)	8 - 106	18 - 76
	[45.04 ± 13.59]	[43.24 ± 13.52]
Mammary gland content ratio (%)	9.31 – 87.60	18.33 – 85.31
	[43.47±16.27]	[51.01 ± 15.48]
System	Canon	
Position	MLO	

CBT, compressed breast thickness; MLO, mediolateral oblique.

### 2.3 Evaluation method

First, we evaluated divergence trends between controls and non-visibles by the overall sample and then by age and CBT based on the average estimated mammary gland content ratio to ensure the appropriate subgroup analysis. P values for paired T-test were calculated through logistic regression analysis. Next, we evaluated the possibility that a mammary gland content ratio ≥50% affects the divergence between controls and non-visibles to specifically identify factors that indicate the possibility of non-visibles. We used a threshold of 50% in this analysis to define a dense breast, which also follows the Japanese guidelines (10). The images were classified into two groups according to their estimated mammary gland content ratios, with a threshold of 50%, and logistic regression analysis was performed to calculate odds ratios and P values for paired T-test between the controls and the non-visibles by the overall sample and then by age and CBT. We used RStudio (version 1.1.456) for the logistic regression analysis.

## 3 Results

**Figure 4:**
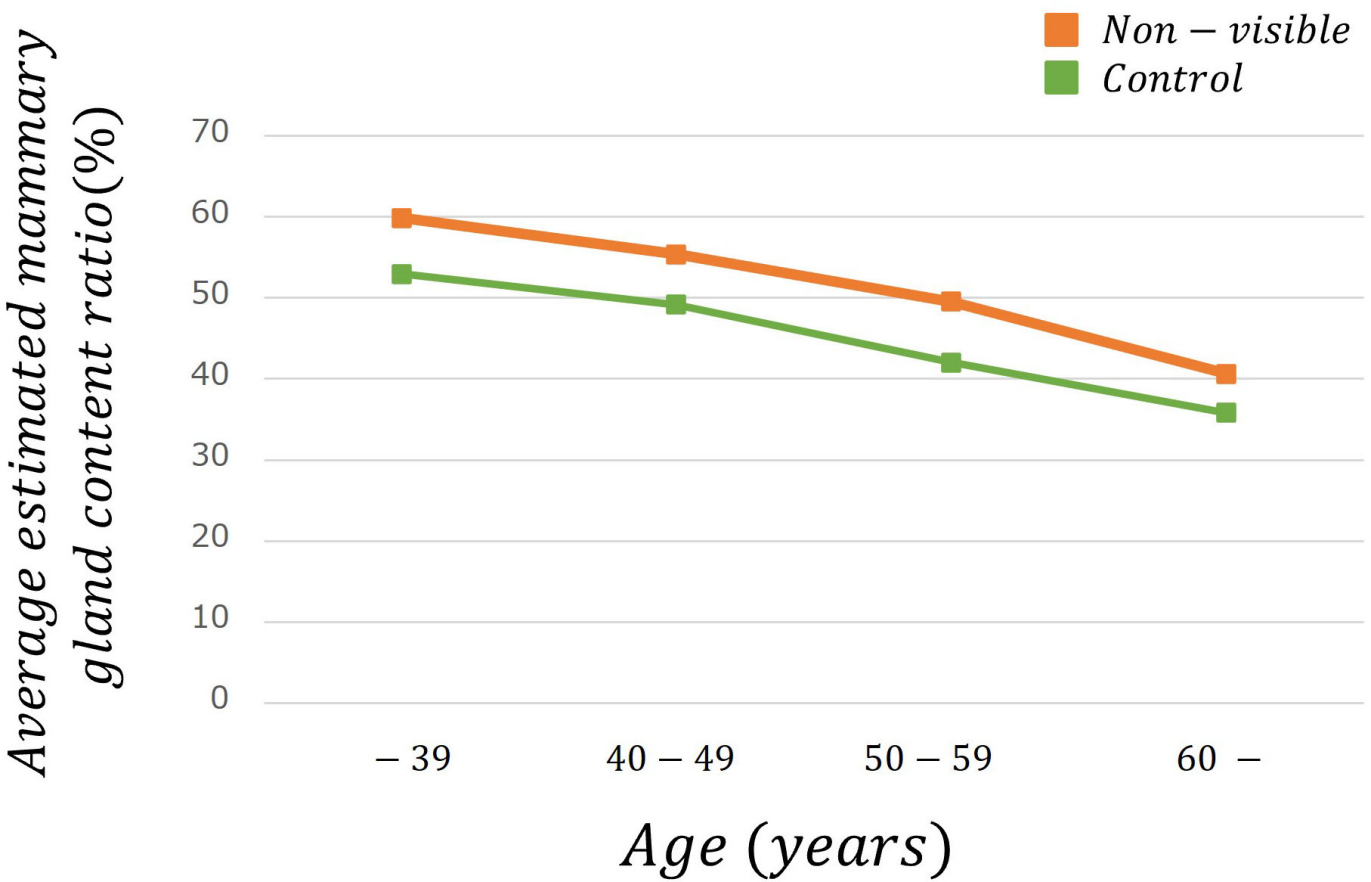
The average estimated mammary gland content ratio among controls and non-visibles by age group.

**Figure 5:**
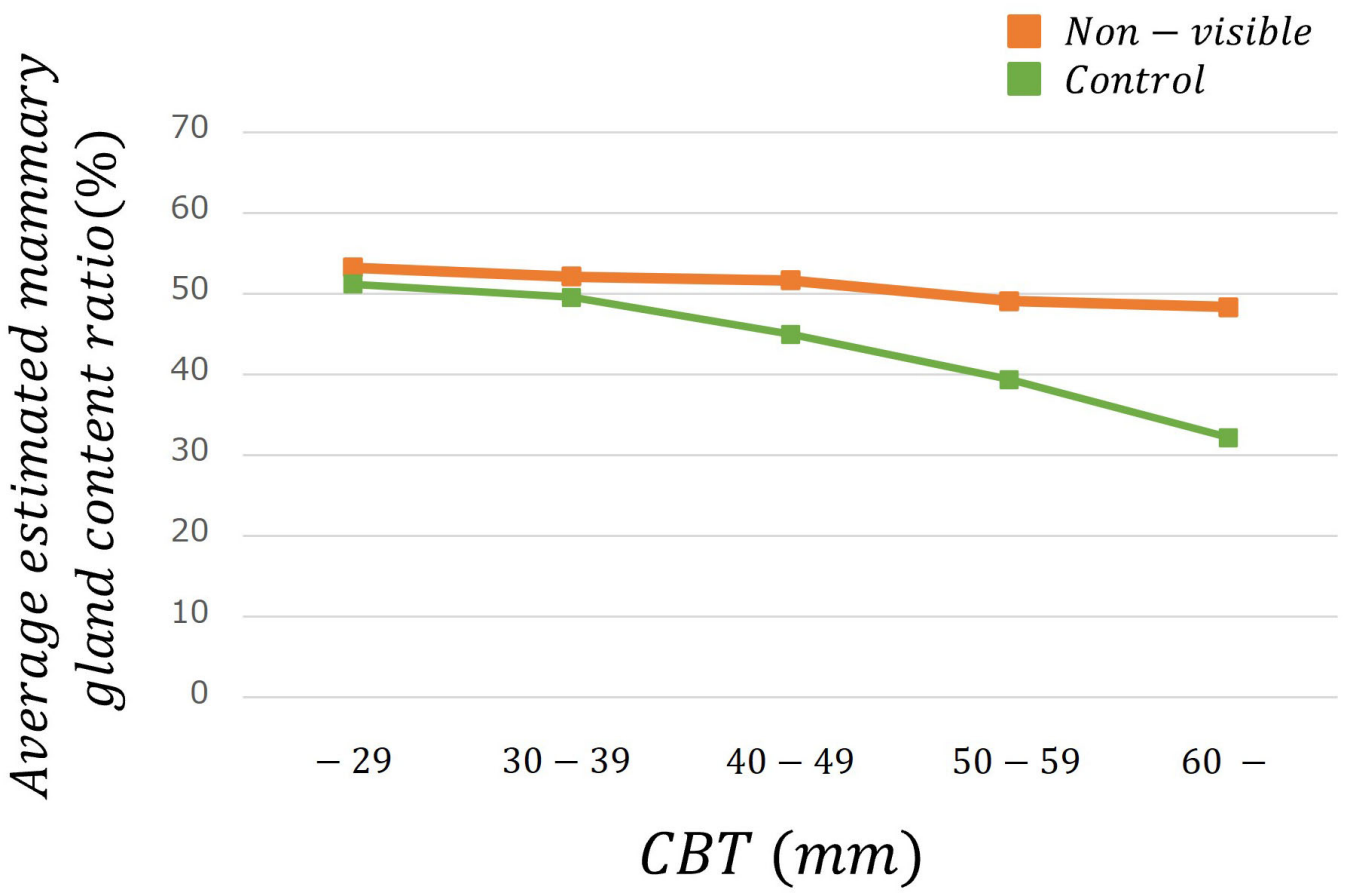
The average estimated mammary gland content ratio among controls and non-visibles by CBT group.

**Table 2** lists the number of images analyzed based on the average estimated mammary gland content ratio. The overall average estimated mammary gland content ratio was significantly higher in non-visibles than in the controls (p < 0.05) (**Table 3**). The difference in the average estimated mammary gland content ratio between the control and non-visible groups was 7.54%. The average estimated mammary gland content ratio was significantly higher in the non-visible group than in the control group when patient age was ≥40 years (p < 0.05) (**Table 3**, **Figure 4**). The difference in the average estimated mammary gland content ratio of the control and non-visible groups for patients aged 40–49 years, 50–59 years, and ≥60 years was 6.20%, 7.48%, and 4.78%, respectively. The average estimated mammary gland content ratio decreased with increasing age in both control and non-visible groups. The average estimated mammary gland content ratio was significantly higher in non-visibles than in controls when the CBT was ≥ 40 mm (p < 0.05) (**Table 3**, **Figure 5**). The difference in the average estimated mammary gland content ratio of the control and non-visible groups for patients with a CBT of 40–49 mm, 50–59 mm, and ≥60 mm was 6.67%, 9.71%, and 16.13%, respectively. In the control group, the average estimated mammary gland content ratio decreased as the CBT increased; however, in the non-visible group, the average estimated mammary gland content ratio was maintained regardless of the CBT. The estimated mammary gland content ratio tended to diverge more between the controls and non-visibles as the CBT increased.

**Table 2:** Number of images in analysis based on the average estimated mammary gland content ratio.

		Number of Images	
		Control	Non-visible
overall		26,232	150
Age (years)	- 39	2,124	15
	40-49	8,573	65
	50-59	8,005	35
	60 -	7,530	35
CBT (mm)	- 29	3,277	19
	30-39	5,900	40
	40-49	7,057	41
	50-59	5,799	34
	60 -	4,199	16

CBT, compressed breast thickness.

**Table 3:** The average estimated mammary gland content ratio in the control and non-visible groups for the overall sample and by age and CBT.

		Average estimated mammary gland content ratio (%)			p-value	OR
		Control	Non-visible	Difference between Controls and Non-visibles		
Overall		43.47	51.01	7.54	**<0.001**	**1.03**
Age (years)	- 39	52.94	59.89	6.95	0.096	1.03
	40-49	49.16	55.36	6.20	**0.002**	**1.03**
	50-59	42.05	49.53	7.48	**0.005**	**1.03**
	60 -	35.84	40.63	4.78	**0.044**	**1.02**
CBT (mm)	- 29	51.16	53.20	2.04	0.531	1.01
	30-39	49.54	52.10	2.56	0.258	1.01
	40-49	44.94	51.62	6.67	**0.005**	**1.03**
	50-59	39.35	49.06	9.71	**<0.001**	**1.04**
	60 -	32.18	48.31	16.13	**<0.001**	**1.06**

CBT, compressed breast thickness; OR, odds ratio.

Figures with p-value ≤ 0.05 are underlined.

**Figure 6:**
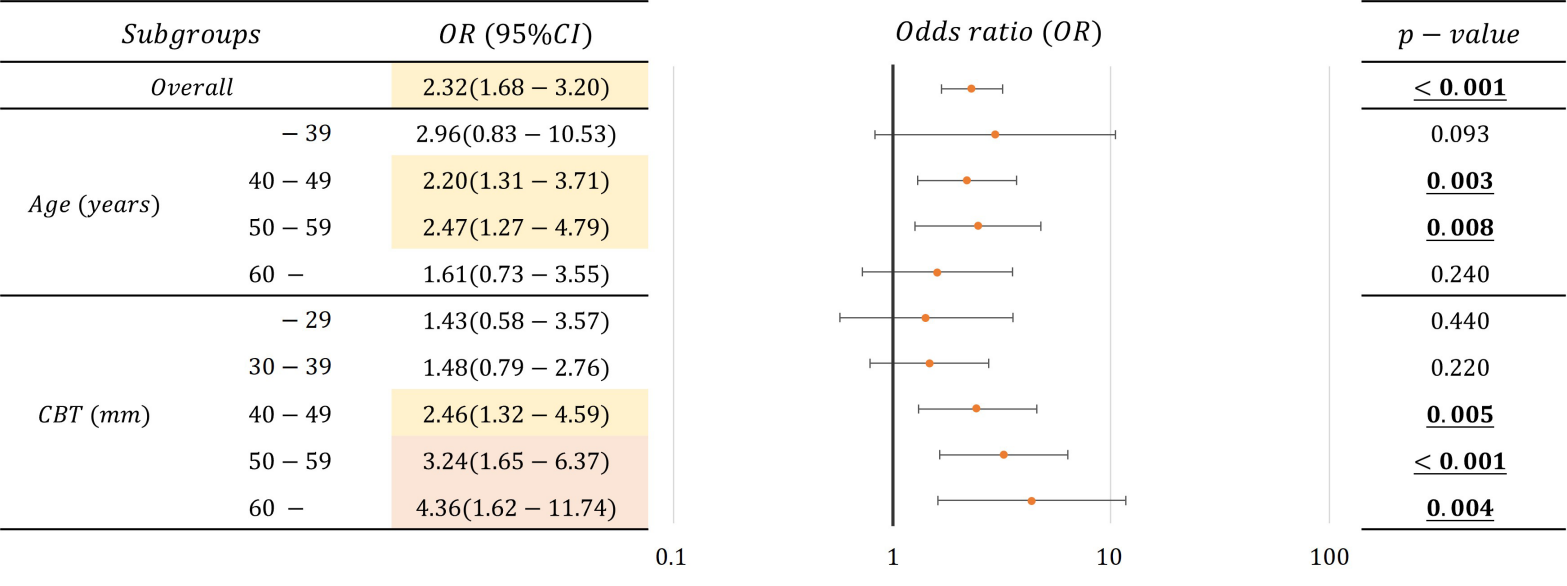
Correlations and odds ratio (OR) about the possibility that mammary gland content ratio ≥50% affects the divergence between controls and non-visibles for the overall sample and by age and CBT (OR < 2.5: Yellow, 2.5 ≦ OR: Red).

**Table 4** lists the number of images analyzed based on a mammary gland content ratio ≥50%. In evaluating the possibility that a mammary gland content ratio ≥50% affects the divergence between controls and non-visibles, positive correlations were observed among non-visibles when the controls were used as baseline (**Figure 6**) (p < 0.05) for the overall sample and for patients aged 40–59 years and those with a CBT ≥40 mm. The corresponding odds ratios were ≥2.20, with a maximum value of 4.36. However, no positive correlation was observed between non-visible findings when using controls as a baseline for patients aged ≤39 years and ≥60 years and for those with a CBT of ≤39 mm.

**Table 4:** Number of images in analysis based on mammary gland content ratio ≥50% groups.

		Threshold of estimated mammary gland content ratio(%)	Number of Images	
			Control	Non - visible
Overall		< 50	17,254	68
		≥ 50	8,978	82
Age (years)	-39	< 50	904	3
		≥50	1,220	12
	40 - 49	< 50	4,391	21
		≥50	4,182	44
	50 - 59	< 50	5,601	17
		≥50	2,404	18
	60 -	< 50	6,358	27
		≥50	1,172	8
CBT (mm)	-29	<50	1,673	89
		≥50	1,604	11
	30 - 39	< 50	32,319	189
		≥50	2,670	22
	40 - 49	< 50	4,483	17
		≥50	2,574	24
	50 – 59	< 50	4,304	16
		≥50	1,495	18
	60 -	< 50	3,563	9
		≥50	636	7

CBT, compressed breast thickness.

## 4 Discussion

We estimated the mammary gland content ratio using an AI system and identified the divergence between controls and non-visibles. We found trends of divergence in the average estimated mammary gland content ratio between controls and non-visibles based on the age and CBT subgroups (**Table 3**, **Figures 4**, **5**). Although the overall average estimated mammary gland content ratio of non-visibles was significantly higher than that of controls, the results of subgroup analysis by age and CBT differed by group. Therefore, it could be possible to differentiate the importance of evaluating the mammary gland content ratio by age and CBT.

Following the result, we identified that an estimated mammary gland content ratio of ≥50% in patients aged 40–59 years or those with ≥40 mm CBT could indicate the possibility of non-visible findings on a mammogram (**Figure 6**). The ratio in the 40–59-year age group showed a significant difference between the controls and non-visibles, which is understandable considering that the lower the age, the higher the mammary gland content ratio. The ratio in patients aged ≤39 years showed no divergence between the controls and non-visibles, which may be related to the small number of cases included in this study. The ratio in patients with a CBT thickness of ≥40 mm showed divergence between the controls and non-visibles, which may be attributed to the hard consistency of breast cancer that makes application of thinner compression challenging as compared with that in controls.

We used an AI system developed using a convolutional neural network that had previously shown a high correlation (17) for estimating the mammary gland content ratio and identifying factors that indicate the possibility of non-visibles. The main problems in clinical research when analyzing big data are that it is time-consuming and involves large inter- and intra-observer variations. The benefits of using AI to address these problems include efficient, quantitative, and objective evaluations. We believe that this is one of the chief reasons for achieving clear results on the relationship of age and CBT with the mammary gland content ratio in this study.

In addition, the subgroups of age and CBT in this study are derived from the DICOM header, allowing for easy acquisition by setting the output parameters to include age and CBT. There are two advantages of using these subgroups. First, age and CBT represent objective measures, as opposed to being derived from questionnaires or other subjective assessments. Second, both age and CBT are integral parameters in nationally recommended breast cancer screening and can be used for a wide range of patients without constraints related to screening methodology. Numerous studies have focused on the relevance of age in this context. For instance, Tran et al. analyzed the association between a family history of breast cancer and breast composition, and the changes in the breast composition of individuals with a family history of breast cancer for the age groups 40–44 years, 45–49 years, and 50–55 years (18). Nara et al. used Volpara, a fully automated volume densitometry program, to identify the best predictors of breast cancer risk during menopause and for age groups with a threshold of 60 years (19). Advani et al. analyzed the association between body mass index (BMI) and breast composition in the age groups of 65–74 years and ≥75 years (20). However, to the best of our knowledge, although there are reports based on BMI (20–23), family history of breast cancer (18), menopausal status (19, 21, 24), microcalcifications (25), benign disease (26), age at menarche and height (27), childbearing history (28), breast cancer subtype (24), endometriosis (29), and skeletal muscle mass index (30), relevant studies considering the CBT are scarce, which is also novel in this regard.

In March 2023, the U.S. Food and Drug Administration mandated the notification of breast composition to patients (31), making it more important than ever to evaluate breast composition during breast cancer screening and routine practice. Further, with the development of genomic medicine in recent years, the screening and treatment modalities have been tailored for individual patients. The results of this study suggest that evaluating breast composition by subgroups, such as age and CBT, may help recommend appropriate testing for individuals. Our findings are therefore clinically relevant for personalized medicine.

For example, our findings may recommend the combined use of mammography and ultrasonography. Combined mammography and ultrasonography increase diagnostic sensitivity but decrease specificity and increase the false-positive rate, which may lead to overdiagnosis (32, 33). In addition, there are no data demonstrating the effect of combined mammography and ultrasonography on reducing breast cancer mortality; therefore, combined use of mammography and ultrasonography is not yet widespread. However, in practice, some cases have non-visibles, which may validate the combined application of mammography and ultrasonography to some extent (13). In this study, we identified factors that indicate the possibility of non-visibles. Therefore, we believe that in the future, it may be possible to suggest patients who may benefit from combined mammography and ultrasonography.

This study had several limitations. As the results of this study are based on data from a Japanese population, they may differ from the findings of Western populations. Additionally, the dataset evaluated in this study was obtained from a single facility, and it is therefore necessary to examine data from multiple facilities. An increased number of non-visibles, may prompt adjustments to the threshold of the mammary grand content ratio, facilitating a more detailed analysis of the data.

In conclusion, we identified factors that indicate the possibility of non-visibles using an AI system developed by us and evaluated the estimated mammary gland content ratio of controls and non-visibles based on age and CBT. The present findings could be used in breast cancer screening and routine practice; they could contribute to the early detection of breast cancer and a reduction in the mortality rate by helping clinicians perform a personalized examination for each patient.

## Data availability statement

The original contributions presented in the study are included in the article/supplementary materials, further inquiries can be directed to the corresponding author.

## Ethics statement

The studies involving humans were approved by the Ethics Committee of Niigata University of Health and Welfare. The studies were conducted in accordance with the local legislation and institutional requirements. The ethics committee/institutional review board waived the requirement of written informed consent for participation from the participants or the participants' legal guardians/next of kin because of research using the opt-out method. Written informed consent was not obtained from the individual(s) for the publication of any potentially identifiable images or data included in this article because of research using the opt-out method.

## Author contributions

CK: Conceptualization, Data curation, Formal Analysis, Funding acquisition, Software, Visualization, Writing – original draft, Writing – review & editing. TO: Investigation, Resources, Validation, Writing – review & editing. MN: Investigation, Validation, Writing – review & editing. SKo: Methodology, Software, Validation, Writing – review & editing. HF: Resources, Writing – review & editing. NK: Supervision, Writing – review & editing. SKa: Conceptualization, Funding acquisition, Project administration, Supervision, Validation, Writing – review & editing.
